# The Chemopreventive Effect of Ginsenoside Compound K Is Regulated by PARP-1 Hyperactivation, Which Is Promoted by p62-Dependent SIRT6 Degradation

**DOI:** 10.3390/nu17030539

**Published:** 2025-01-31

**Authors:** Sang-Hun Kim, Sung-Hwan Ki, Seok-Woo Hyeong, Seon-Hee Oh

**Affiliations:** 1 Department of Anesthesiology and Pain Medicine, School of Medicine, Chosun University, 309 Pilmundaero, Dong-gu, Gwangju 61452, Republic of Korea; ksh3223@chosun.ac.kr; 2 College of Pharmacy, Chosun University, 309 Pilmundaero, Dong-gu, Gwangju 61452, Republic of Korea; shki@chosun.ac.kr; 3 Department of Biomedical Sciences, Graduate School of Chosun University, 309 Pilmundaero, Dong-gu, Gwangju 61452, Republic of Korea; gudtjrdn11@naver.com; 4 School of Medicine, Chosun University, 309 Pilmundaero, Dong-gu, Gwangju 61452, Republic of Korea

**Keywords:** ginsenoside compound K, lung cancer, p62 protein, PARP-1 protein activation, SIRT6 protein

## Abstract

Background and aims: Ginsenoside compound K (CK), a saponin metabolite of ginseng, exerts anticancer effects; however, its molecular mechanisms of action in lung cancer remain unclear. We investigated the involvement of silent information regulator 6 (SIRT6) and poly (ADP-ribose) polymerase 1 (PARP-1) in the anticancer effects of CK in lung cancer. Methods and Results: CK induced PARP-1 activation-mediated parthanatos via sequestosome-1/p62-mediated SIRT6 degradation and inhibited the proliferation of H460 cells. Although CK reduced procaspase-8 levels, no significant apoptotic cleavage of procaspase-3 or PARP-1 was observed. Furthermore, CK upregulated p27, p21, phospho-p53, and gamma-H2AX levels. CK increased LC3-II levels in a p62-independent manner, but p62 was upregulated by autophagy inhibition, indicating that p62 is involved in CK-induced autophagy. CK-treated cells showed typical features of parthanatos, including PARP-1 hyperactivation, intracellular redistribution of poly ADP-ribose and pro-apoptotic factors, and chromatin fragmentation. SIRT6 was degraded in a CK concentration- and time-dependent manner. SIRT6 protein was upregulated by PARP-1 inhibition, nicotinamide adenine dinucleotide (NAD)+ supplementation, antioxidants, and p62 knockdown, but was decreased by autophagy blockade. PARP-1 activation was negatively correlated with SIRT6 levels, indicating that SIRT6 and PARP-1 activation play complementary roles in CK-induced growth inhibition. Immunofluorescence staining, fractionation studies, and immunoprecipitation were used to confirm the colocalization and interaction between p62 and SIRT6. Conclusions: PARP-1 activation is promoted by p62-mediated SIRT6 degradation, which plays an important role in CK-induced growth inhibition. Therefore, SIRT6 is a potential biomarker for the chemopreventive effect of CK in lung cancer cells, but further studies on SIRT6 are needed for the clinical application of CK.

## 1. Introduction

Ginseng (*Panax ginseng* C. A. Meyer) has been used as a medicinal plant in Asia, and the efficacy of red ginseng produced by the steam process is well known. During the production process of red ginseng, the sugars of major ginsenosides, including Rb1, Rb2, Rc, Rd, Re, Rf, and Rg1, are hydrolyzed to produce minor ginsenosides such as compound K (CK), Rd, Rg3, Rc, Ra1, Ra2, and Ra3; in particular, CK is produced through biotransformation by human intestinal bacteria [1,2]. Ginsenosides are poorly absorbed, resulting in low efficacy and difficult clinical application. CK has attracted great interest in the medical and pharmaceutical fields due to its relatively high absorption rate, exhibiting a variety of potential pharmacological activities, including chemoprevention, antioxidant, antidiabetic, and neuroprotective properties [2,3].

Lung cancer ranks second in global cancer prevalence and mortality. Several chemopreventive agents have shown promise in reducing the risk of lung cancer; however, their clinical effectiveness remains unknown [4]. Therefore, an in-depth understanding of the molecular mechanisms underlying lung cancer is necessary for the development of effective treatments.

Silent information regulator 6 (SIRT6) acts as a nicotinamide adenine dinucleotide (NAD+)-dependent deacetylase and mono-ADP-ribosyltransferase [5]. It also plays an important role in genome maintenance, which is key to biological processes such as aging, apoptosis, and tumorigenesis, and is localized in the nucleus [6,7]. Under stress conditions, SIRT6 migrates to the cytoplasm and participates in stress granule formation, thereby promoting cell survival [8]. In addition, when in the endoplasmic reticulum (ER), SIRT6 can modulate inflammation through tumor necrosis factor-α release [9]. SIRT6 can act as a tumor promoter or suppressor, and its expression is downregulated in some cancers, including hepatocellular carcinoma [10], colorectal cancer [11], and head and neck squamous cell carcinoma [12], and is associated with the inhibition of apoptosis. In contrast, SIRT6 overexpression in breast cancer cells reinforces resistance to anticancer drugs such as paclitaxel and epirubicin [13]. Moreover, many types of cancer cells, including H460, A549, and prostate cancer cells, have high levels of SIRT6, and inhibiting SIRT6 in these cells results in cell death [14,15]. These contrasting findings may be attributed to the cell-type specificity and diverse functions of SIRT6. Further studies are essential to elucidate the function of SIRT6 under different pathological conditions.

Poly (ADP-ribose) polymerase 1 (PARP-1) is the most well-known PARP family member. When DNA is damaged, PARP-1 catalyzes the transfer of ADP-ribose units from NAD+ to itself or other proteins, and these post-translational modification plays an important role in repairing DNA damage [16]. Large-scale DNA damage results in PARP-1 hyperactivation through poly ADP ribosylation (PARylation), which reduces cellular ATP levels and ultimately leads to parthanatos [17]. In addition, when severe DNA damage occurs, PARP-1 is cleaved into two fragments (89 and 24 kDa) that are enzymatically inactivated by caspases-3 and -7, inducing cell death [18]. SIRT6 promotes DNA repair by catalyzing the PARylation of PARP-1 [19]. Therefore, to better understand the role of SIRT6 in pathophysiology, the relationship between SIRT6 and PARP-1 needs to be elucidated.

CK has been reported to exhibit significant anticancer effects in lung cancer cells [20]. CK induces ER stress by mediating STAT3 phosphorylation and Ca^2+^ release in human lung cancer cells [21,22]. Reactive oxygen species (ROS) have an important role in CK-induced apoptosis by way of its mitochondrial caspase-dependent signaling in HT-29 and H460 cells [23,24]. Furthermore, CK elicits autophagy and apoptosis through AMP-kinase-dependent activation of mammalian target of rapamycin and the c-Jun N-terminal kinase signaling pathways in the A549 and H1975 cells [25]. However, the molecular mechanisms underlying the antitumor effects of CK remain unclear. Ginsenoside Rc prevents nonalcoholic fatty liver disease by increasing the deacetylase activity of SIRT6 [26]. Ginsenoside Rb3 reduced apoptosis and inflammation in palmitate-treated podocytes by upregulating SIRT6 and peroxisome proliferator-activated receptor delta (PPARδ) levels [27]. High cytoplasmic SIRT6 expression is strongly implicated in poor prognosis and diminished chemosensitivity in lung cancer patients [28]. Although CK induces apoptosis in lung cancer cells, the involvement of SIRT6 in this process remains unclear. Therefore, further studies are warranted to elucidate the mechanism by which CK exerts its chemopreventive effects.

This work investigated the involvement of SIRT6 and PARP-1 in the anticancer effects of CK in lung cancer. These effects are regulated by partanatos via PARP-1 hyperactivation and facilitated by p62-activated SIRT6 degradation.

## 2. Materials and Methods

### 2.1. Reagents and Antibodies

Compound K was purchased from Ambo Institute (Daejeon, Republic of Korea). Chloroquine (CQ), bafilomycin A1 (BaF1), 3-(4,5-dimethylthiazol-2-yl)-2,5-diphenyltetrazolium bromide (MTT), 4-amino-1,8-naphthalimide (ANI), 3-amino benzamide (3-AB), leptomycin B (LMB), and anti-β-actin antibody were acquired from Sigma-Aldrich (St. Louis, MO, USA). Apoptosis-inducing factor (AIF), ubiquitin- and rhodamine-conjugated goat anti-rabbit antibodies, and Hoechst-33342- and FITC-conjugated goat anti-mouse antibodies were percussed at Santa Cruz Biotechnology (Santa Cruz, CA, USA). PARP-1, procaspase-3, procaspase-8, phospho-p53, LC3B, p27, p21, SIRT6, poly ADP-ribose, and gamma-H2AX antibodies were percussed at Cell Signaling Technology (Beverly, MA, USA). The SQSTM1/p62 antibody and zVAD-FMK were acquired from Abnova (Taipei City, Taiwan) and MBL (Woburn, MA, USA), respectively.

### 2.2. Cell Culture

H460 human lung cancer cells (HTB-177™) were grown in Dulbecco’s modified Eagles’ medium (Wellgin, Gyeongsan, Republic of Korea) supplemented with 10% fetal bovine serum (Welgene) and penicillin–streptomycin (Welgene). The cells were grown at 37 °C in a humidified incubator containing 5% CO_2_. Cell lines were checked and determined to be free of mycoplasma and were used between passages 3 and 12.

### 2.3. Cytotoxicity Assays

Cell viability was assessed by MTT assay. Cell suspensions (200 μL, 1 × 10^5^ cells/mL) were seeded in 48-well plates and cultured for 2 days, then treated with drugs as described in the figure legend and incubated in MTT solution (0.5 mg/mL) for 2 h at 37 °C. Formazan crystals were solubilized in dimethyl sulfoxide and the absorbance of the solution was measured at 540 nm by using a microplate reader (Perkin-Elmer, Waltham, MA, USA). Experiments were replicated a minimum of three times. The values are given as mean ± standard deviation (SD) of magnification increase over control.

### 2.4. RNA Interference and Overexpression

The target-specific siRNAs against ATG5, SQSTM1, and SIRT6 mRNAs were generated by Genolution (Seoul, Republic of Korea). Cells were transfected with target-specific siRNAs or control siRNAs (Thermo Fisher Scientific, Waltham, MA, USA) using LipofectamineTM RNAiMAX reagent (Invitrogen, Carlsbad, CA, USA). pcDNA3.1-Sirt6 was kindly gifted by Dr. Byung-Hyun Park (Chonbuk National University, Jeonju, Republic of Korea). Cells were transfected with plasmid DNA (0.5 μg) or an empty vector using X-tremeGENE HP-DNA transfection reagent (Roche Diagnostics, Mannheim, Germany) following the supplier’s protocol.

### 2.5. Immunoblotting and Immunoprecipitation (IP)

Cells were lysed in lysis buffer that included a protease cocktail (Roche Applied Science, Penzberg, Germany). Proteins (15–35 μg) were resolved by 10–12% SDS-PAGE and transferred to polyvinylidene difluoride (PVDF) membranes (Millipore, Bedford, MA, USA), which were then blocked with 5% skim milk (BioShop Canada Inc., Burlington, ON, Canada) and incubated with the primary and secondary antibodies. Chemiluminescent substrate (Millipore) was employed to visualize the protein. The semi-quantification of protein levels was conducted with Image J software Java 1.8.0_112 [64-bit] (National Institutes of Health, Bethesda, Rockville, MD, USA). Densitometry data for each blot are shown in the corresponding Supplementary File (Figure S5). For IP, cells were lysed, and 800 μg of total protein was used as previously described [29].

### 2.6. Immunofluorescence (IF)

Cells were grown on coverslips in 12-well plates, treated with drugs as described in the figure legend, washed with phosphate-buffered saline (PBS), and fixed on ice for 10 min in neutral buffered formalin. IF staining was performed as previously described [29]. Briefly, drug-treated cells were rinsed with PBS and incubated with 0.05% Triton X-100 for 20 min. After rinsing with PBS, cells were then blocked with 2% bovine serum albumin (BioShop Canada Inc.). Cells were subsequently incubated with primary antibody and fluorescence-conjugated secondary antibody. The nuclei were counterstained with Hoechst 33,342 (1 μg/mL), mounted using Simpo-mount (GBI Labs,, Bothell, WA, USA), and images were acquired using a Nikon Eclipse TE300 Fluorescent Microscope (Nikon, Tokyo, Japan).

### 2.7. Subcellular Fractionation

After CK treatment, the cells were harvested, washed with PBS, and fractionated as previously described [29]. Briefly, cell pellets were suspended in hypotonic buffer (20 mM HEPES-KOH, pH 7.0, 10 mM KCl, 1.5 mM MgCl2, 1 mM sodium EDTA, 1 mM EGTA, 250 mM sucrose) and contained in cOmplete™ Mini EDTA-free protease inhibitor (Roche Ap-plied Science). The cells were then passed through a needle to homogenize them and centrifuged at 800× *g* for 4 min. The resulting pellet was the nucleus-enriched insoluble fraction. The supernatant, enriched in cytoplasm and organelles, was then centrifuged at 10,000× *g* for 20 min at 4 °C. The supernatant and organelle-enriched pellet were used as the soluble and particulate fractions, respectively.

### 2.8. Statistical Analysis

All experiments were performed independently at least three times. Data are presented as the mean ± SD. The statistical significance of the differences among the groups was determined using one-way analysis of variance (*p* < 0.05).

## 3. Results

### 3.1. CK Induces Cellular Morphological Changes and Inhibits Cell Proliferation

**Figure 1:**
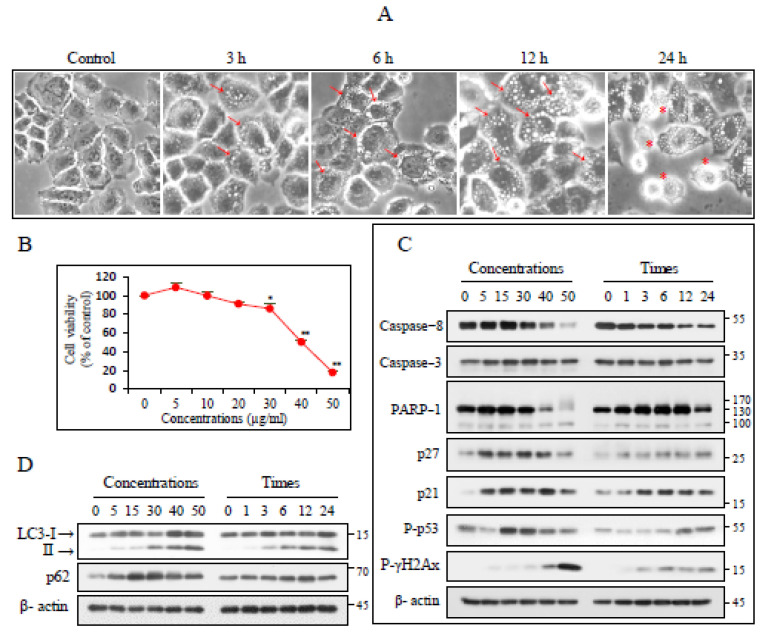
CK induced morphological changes and cell proliferation inhibition. (**A**) H460 cell morphological changes after the exposure to increasing CK concentrations for up to 24 h monitored by phase-contrast microscopy. Arrows and red asterisk denote the cytoplasmic vacuoles and floating cells, respectively. (**B**) Cells were treated as outlined in A, and cell viability was determined using an MTT assay. Data are expressed as the mean ± SD. * *p* < 0.05; ** *p* < 0.005. (**C**,**D**) Cells were exposed for 18 h in ascending order of CK concentration or for up to 24 h with a CK concentration of 35 µg/mL. Lysates were analyzed based on immunoblotting. β-actin was used as a loading control (*n* > 3).

CK induced morphological changes in H460 cells, including cytoplasmic vacuole formation, starting at 3 h after treatment. The number and size of the vacuoles increased continuously before the cells detached from the culture dish (Figure 1A). The sensitivity to CK was analyzed using an MTT assay 24 h after treatment. The CK half-maximal inhibitory concentration (IC50) was approximately 37 µg/mL (Figure 1B). The expression of apoptosis-related proteins was explored to identify the signaling pathways underlying sensitivity to CK. CK exposure decreased the caspase-8 levels but did not affect those of caspase-3; at high CK concentrations (40 and 50 µg/mL), the 116 kDa PARP-1 protein levels were markedly reduced. However, no significant PARP-1 apoptotic cleavage was observed. Furthermore, CK upregulated p27, p21, phospho-p53, and gamma-H2AX levels, the last of which is a double-stranded DNA break indicator. These results indicate that CK inhibited cell growth through caspase-independent cell-death pathways and DNA damage mediated cell-cycle arrest (Figure 1C). CK increased the levels of the autophagy marker LC3-II in a concentration- and time-dependent manner. However, the levels of the autophagy adapter p62 did not decrease significantly (Figure 1D). These results indicate that various signaling pathways are involved in the CK-induced inhibition of cell proliferation.

### 3.2. CK Induces Parthanatos Through PARP-1 Activation and SIRT6 Degradation

**Figure 2:**
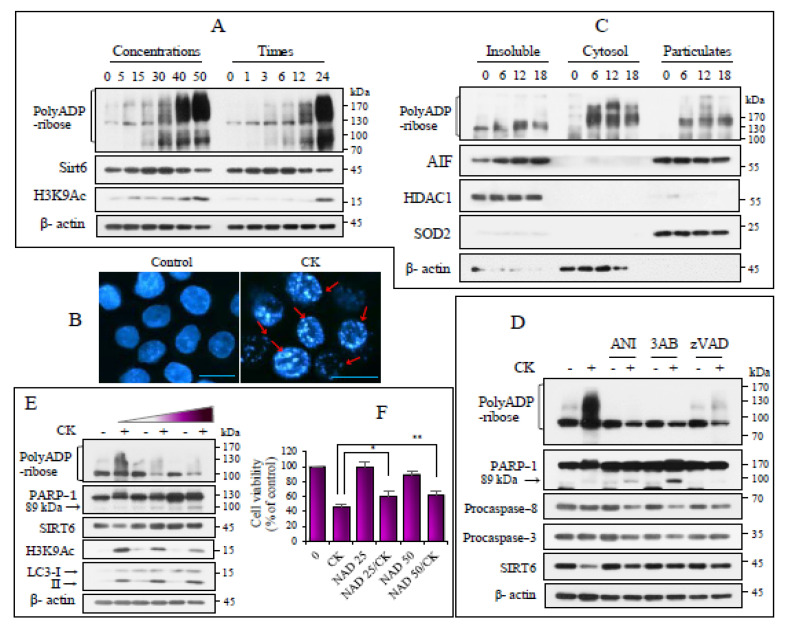
CK induced parthanatos and SIRT6 protein degradation. (**A**) H460 cells were exposed for 18 h in ascending order of CK concentration or for up to 24 h with a CK concentration of 35 µg/mL. Lysates were analyzed based on immunoblotting. β-actin was used as a loading control (*n* > 3). (**B**) H460 cells cultured on coverslips were exposed to CK for 18 h, fixated, and the nuclei were stained with Hoechst 33342. Arrows indicate the parthanatos nuclear features. Images were taken using a fluorescence microscope (×200). (**C**) Cells were exposed to CK for 6, 12, and 18 h and subsequently performed intracellular fractionation into insoluble (nuclear enriched membrane), cytosolic, and particulate (mitochondrial enriched) fractions. The enrichment of each fraction was assessed via immunoblotting for HDAC1, β-actin, and SOD2 (*n* > 3). (**D**) Cells were exposed to CK for 18 h after a pretreatment with 3-AB, ANI, zVAD-fmk, or dimethylsulfoxide (DMSO) control for 2 h. Lysates were analyzed using immunoblotting for the indicated proteins. β-actin was used as the loading control. (**E**,**F**) Cells were exposed to increasing NAD+ concentrations (25 and 50 nM) for 18 h after pretreatment and then to CK. Immunoblotting was used to analyze the levels of the indicated proteins. Cells were cultured for 24 h as outlined in E, and cell viability was measured using MTT. Data are presented as mean ± SD. * *p* < 0.05; ** *p* < 0.005.

Poly (ADP-ribose) was deposited in a concentration- and duration-dependent manner, which started to increase at 3 h post treatment. PARP-1 activation decreased after 24 h of CK exposure, accompanied by PARP-1 cleavage and decreased procaspase-3 levels (Figure 2A and Figure S1). SIRT6 was highly expressed in H460 cells but poorly expressed at high CK concentrations (≥40 μg) or after 12 h of CK treatment. H3K9Ac levels increased after prolonged CK exposure (Figure 2A and Figure S1), indicating that CK inversely regulated PARP-1 activation and SIRT6 function.

As PARP-1 overactivation leads to parthanatos, CK-induced morphological changes were observed in the treated cells. Chromatin constriction and pronounced chromatin fragmentation arrayed in loops along the nuclear membrane (a typical feature of parthanatos) were observed, which differed from nuclear apoptotic changes (Figure 2B). To obtain evidence of parthanatos, cells exposed to CK subfracted into insoluble, soluble, and particulate fractions, and the enrichment of each fraction was assessed using corresponding protein markers. After CK treatment, free poly (ADP-ribose) was confined to the cytosolic portion, and lesser amounts were detected in the insoluble and microparticulate fractions. AIF levels progressively decreased in the mitochondrial enriched fraction and increased in the nuclear enriched subfractions (Figure 2C), indicating an association between CK-induced growth inhibition and parthanatos.

We investigated the relationship between SIRT6 and PARP-1 in CK-exposed cells. PARP-1 activation is attenuated by PARP inhibitors, including 3-AB, ANI, and the pan-caspase inhibitor zVAD-fmk. PARP inhibition induced by zVAD-fmk generated a pro-apoptotic cleavage fragment of 89 kDa and reduced caspase-8 and -3 protein levels compared to those found in CK-exposed cells. In addition, PARP-1 inhibition reversed the CK-induced decrease in SIRT6 expression (Figure 2D). Since NAD+ levels affect PARP-1 activation and SIRT6 function, the effects of CK on these proteins were confirmed using NAD+. Exogenous NAD+ supplementation significantly inhibited CK-induced PARylation and induced PARP-1 cleavage in a concentration-dependent manner while downregulating CK-induced H3K9Ac levels via SIRT6 protein upregulation. Additionally, PARP-1 inhibition increased autophagy (Figure 2E). NAD+ supplementation significantly increased, but did not fully restore cell viability, possibly because PARP-1 inhibition induced apoptotic cleavage of PARP-1 (Figure 2F). These results suggest that PARP1 activation and SIRT6 activity play reciprocal roles in CK-induced cell growth inhibition.

### 3.3. SIRT6 Modulates the Biological Function of PARP-1

**Figure 3:**
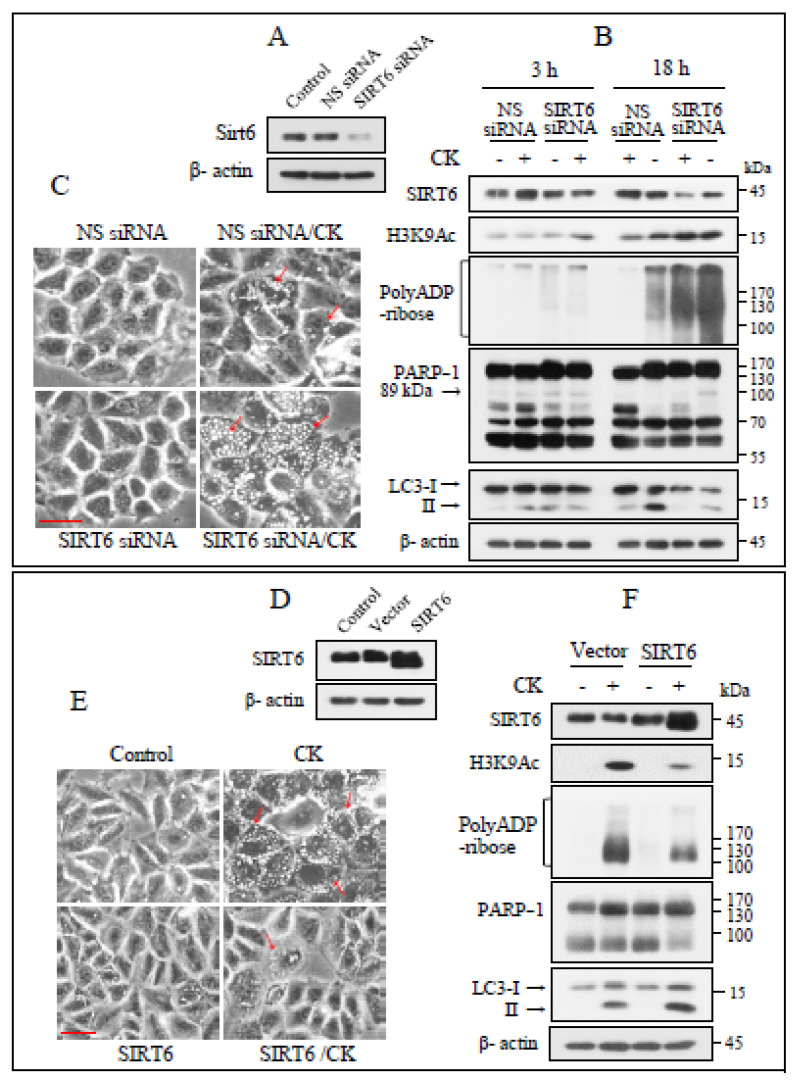
SIRT6 negatively regulates PARP-1 activation by CK. (**A**) Immunoblotting was used to evaluate the knockdown efficiency of Sirt6-specific siRNA. NC = negative control. (**B**) H460 cells infected with NC or Sirt6 siRNA were challenged with CK for 18 h and the levels of the shown proteins were assayed using immunoblotting (*n* = 3). (**C**) Morphological changes in cells treated as in B were viewed with phase-contrast microscopy. Arrows represent cytoplasmic vacuoles. (**D**) After 24 h of transfection with an empty vector or pcDNA3.1-Sirt6, immunoblotting for SIRT6 was performed. (**E**,**F**) Cells overexpressing SIRT6 were treated with 35 µg/mL of CK for 18 h, observed for morphological changes, and then harvested and lysed. Immunoblotting was used to analyze the levels of the indicated proteins. β-actin was taken as a loading control. Arrows indicate the cytoplasmic vacuoles. Scale bar = 25 µm.

The expression of PARP-1 and SIRT6 was inversely regulated by CK. To investigate this further, we knocked down the SIRT6 gene by infusing SIRT6 siRNA and performed immunoblotting analysis (Figure 3A). SIRT6 knockdown cells were challenged with CK for 3 and 18 h. After 3 h of treatment, PARylation and H3K9Ac levels were slightly upregulated and were markedly enhanced at 18 h post treatment. SIRT6 knockdown also induced PARP-1 cleavage and reduced autophagy (Figure 3B). At 18 h post treatment, the number of cytoplasmic vacuoles induced by CK exposure was higher in SIRT6 knockdown cells than in wild-type cells (Figure 3C).

We then investigated the role of SIRT6 overexpression in H460 cells (Figure 3D); in SIRT6-overexpressing cells, CK-induced cytoplasmic vacuolation (Figure 3E), poly (ADP-ribose), and H3K9Ac levels were reduced, whereas PARP-1 cleavage was not induced and autophagy was increased (Figure 3F). Therefore, CK-induced PARylation levels are inversely correlated with SIRT6 protein and autophagy levels, and SIRT6 plays a critical role in the biological function of PARP-1.

### 3.4. CK Regulates SIRT6 and PARP-1 Activation Through Oxidative Stress and Autophagy

**Figure 4:**
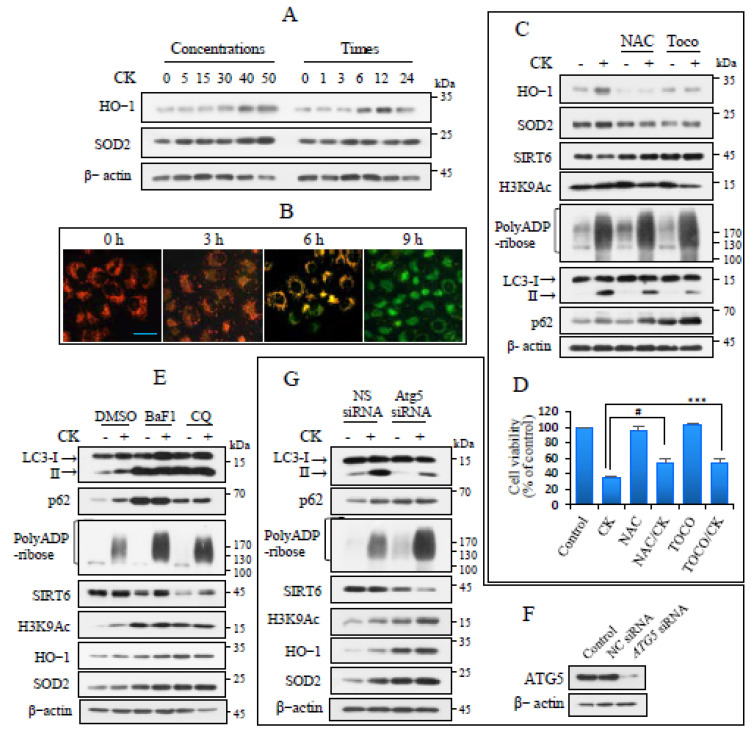
Oxidative stress and autophagy regulate CK-induced PARP-1 activation and SIRT6 protein stability. (**A**) H460 cells were exposed for 18 h in ascending order of CK concentration or for up to 24 h with a CK concentration of 35 µg/mL. Lysates were analyzed based on immunoblotting. (**B**) Cells were exposed to CK a maximum of 8 h and were stained with JC-1 dye. Images were captured under a fluorescence microscope. Scale bar = 25 µm. (**C**) Cells were pretreated with NAC (5 mM) or tocopherol (25 µM) for 2 h and then continuously treated with CK for 18 h. Immunoblotting was used to analyze the levels of the indicated proteins (*n* ≥ 3). (**D**) After culturing cells treated as described in C for 24 h, the viability was determined using the MTT assay. Data are expressed as the mean ± SD. # *p* < 0.001; *** *p* < 0.0005. (**E**) Cells were pretreated with BaF1 (100 nM), CQ (50 µM), and DMSO for 2 h and then exposed to CK for 18 h. The lysates were subjected to immunoblotting for the indicated proteins. (**F**,**G**) The knockdown efficiency obtained using ATG5-specific siRNA was evaluated using immunoblotting for ATG5. NC = negative control. Cells knocked down with NC or ATG5 siRNA were exposed to CK (35 µg/mL) for 18 h and the levels of each protein were assayed using immunoblotting. β-actin was taken as a loading control (*n* = 3).

Heme oxygenase (HO)-1 and superoxide dismutase 2 (SOD2) levels increased in a concentration- and time-dependent manner (Figure 4A), suggesting that CK treatment produced ROS. CK-induced oxidative stress was associated with mitochondrial damage, as demonstrated by mitochondrial membrane potential changes observed using JC-1, a selective mitochondrial membrane potential probe. The control cells exhibited red fluorescence; however, after 9 h of CK exposure, most of the cells emitted green fluorescence (Figure 4B). The effects of oxidative stress on SIRT6 function and PARP-1 activation were examined after treatment with antioxidants before CK treatment. Antioxidant treatment reduced CK-induced HO-1 and SOD2 levels and increased SIRT6 levels, which had been decreased by CK treatment alone. Additionally, the antioxidants increased CK-induced PAR and p62 levels, thereby reducing autophagy (Figure 4C). Antioxidant treatment significantly increased but did not completely restore CK-induced cell viability, likely because of high PARP-1 activation (Figure 4D).

Given oxidative stress is an important factor in autophagy induction, we investigated the effects of autophagy on PARP-1 activation and SIRT6 function during CK exposure. Autophagic flux was blocked using pharmacological autophagy inhibitors, including BaF1 and CQ. The effective blockade of autophagy was confirmed by LC3-II and p62 accumulation. The inhibition of autophagy further increased CK-induced poly (ADP-ribose), H3K9Ac, HO-1, and SOD2 levels and decreased SIRT6 levels (Figure 4E). These results were further confirmed through genetic manipulation by transfecting H460 cells with an siRNA targeting the autophagy-related gene ATG5 (Figure 4F). Inhibiting autophagosome formation decreased CK-induced LC3-II and p62 protein levels, leading to downstream signaling results similar to those obtained using pharmacological autophagy inhibitors (Figure 4G). These results suggest that CK-induced autophagy is involved in the attenuation of PARP-1 activation, whereas oxidative stress regulates the stability of SIRT6.

### 3.5. Cytoplasmic Translocation of SIRT6 Depends on p62

**Figure 5:**
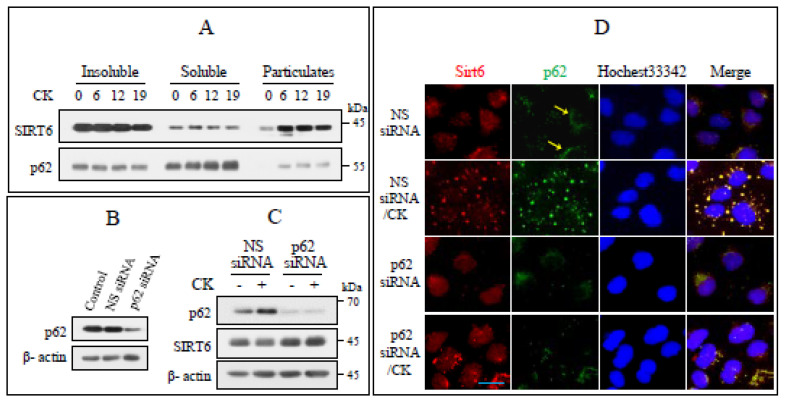
The subcellular translocation of SIRT6 depends on p62. (**A**) The indicated proteins were analyzed in the subcellular fraction samples obtained in Figure 2C. (**B**,**C**) Immunoblotting for p62 was performed to assess the knockdown efficiency of the p62-specific siRNA. NC = negative control. Cells transfused with NC or p62 siRNA were incubated with CK (35 µg/mL) for 18 h. The shown proteins were assayed by immunoblotting. β-actin was taken as a loading control (*n* = 3). (**D**) Cells grown on coverslips were transfected with NC or p62 siRNA, exposed to CK for 12 h, fixed, and performed IF immunostaining for both SIRT6 (red) and p62 (green). Nuclei were counterstained with Hoechst 33342 (blue). The yellow arrows indicate p62 in the perinuclear region. Scale bar, 25 µm.

The subcellular localization of SIRT6 and p62 was evaluated based on the fractionation study described above (Figure 2C). SIRT6 was mainly confined to the nuclear concentrated insoluble fraction of untreated cells and the particulate fraction after CK treatment. Moreover, p62 was localized in the soluble and insoluble fractions of untreated cells. In contrast, after CK exposure, p62 was found mainly in the soluble fraction and to a smaller extent in the microparticulate fraction (Figure 5A). To investigate the interaction between SIRT6 and p62, we exposed p62 knockdown cells to CK. p62 knockdown increased SIRT6 levels following CK exposure (Figure 5B,C). This was validated by IF staining with the use of anti-p62 and anti-SIRT6 antibodies. In non-treated control cells, SIRT6 was detected in the nucleus. Following CK exposure, weak and diffuse SIRT6 staining was observed in the nucleus and puncta of varying sizes were observed in the cytoplasm. p62 was mainly localized in the perinuclear region of non-treated control cells (arrows). After CK treatment, p62 staining was observed as diffusely distributed puncta of varying sizes and as fluorescence in the cytoplasm. Furthermore, the p62 and SIRT6 puncta entirely overlapped: when p62 was knocked down, SIRT6 was noticeably localized to the nucleus (Figure 5D). These results suggest that the trafficking of nuclear SIRT6 to the cytoplasm is p62-dependent.

### 3.6. SIRT6 Interacts with p62 in Response to CK Treatment

**Figure 6:**
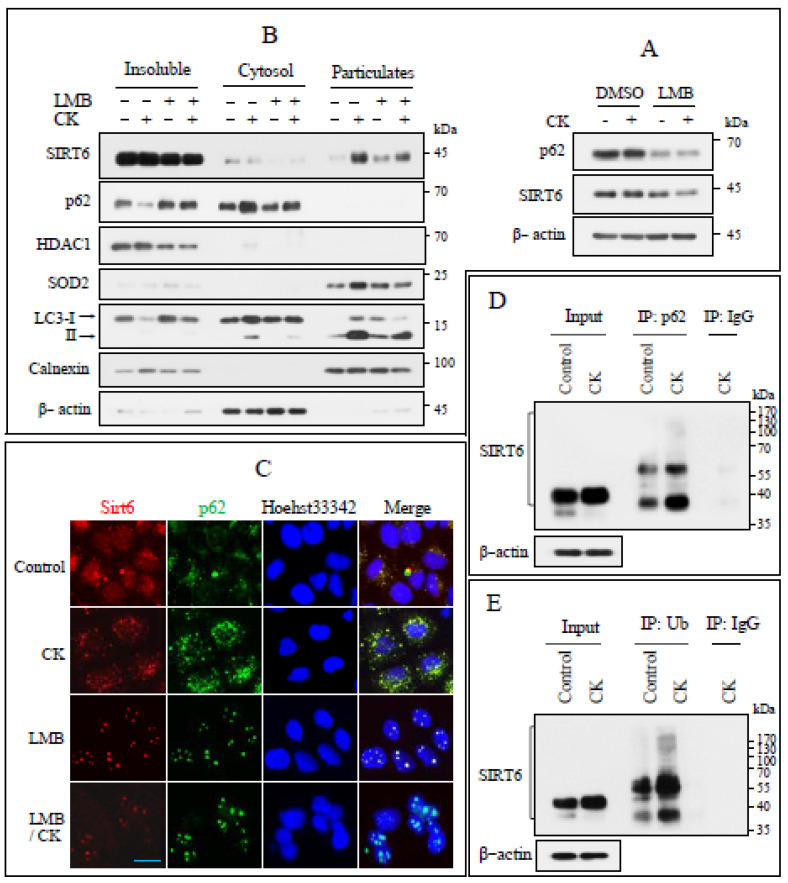
SIRT6 can interact with p62 through its ubiquitination. (**A**) H460 cells were pretreated with LMB (25 nm) or DMSO for 2 h and then continuously treated with CK for 18 h. (**B**) Cells were harvested and subjected to intracellular fractionation into insoluble (nuclear enriched membrane), cytosolic, and microparticulate fractions. Enrichment of individual fractions was measured using immunoblotting for HDAC1 (nucleus), β-actin (cytosol), SOD2 (mitochondria), LC3-II (autophagosome), and calnexin (endoplasmic reticulum) (*n* > 3). (**C**) Cells grown on coverslips were treated with DMSO or LMB (25 nm) for 2 h, continuously exposed to CK for 12 h, fixed, and performed IF staining for SIRT6 (red) and p62 (green). Nuclei were counterstained by Hoechst 33342 (blue). Scale bar, 25 µm. (**D**,**E**) Cells were exposed to CK (30 µg/mL) for 12 h, lysates were immunoblotted for SIRT6, and 800 µg of the residual protein was used for IP assays using p62, ubiquitin (Ub), and mouse IgG antibodies, followed by immunoblotting for SIRT6 (*n* = 2).

Treatment with LMB, a specific nuclear export inhibitor, reduced the levels of p62 and SIRT6 proteins induced by CK treatment (Figure 6A). CK-treated cells were fractionated into insoluble (nucleus-enriched membranes), soluble (cytosol), and particulate (mitochondria, autophagosomes, and ER) fractions, the enrichment of which was confirmed by immunoblotting using specific protein markers. LMB treatment reduced the translocation of SIRT6 and p62 to the particulate and cytosolic fractions, respectively (Figure 6B). The cells were then exposed with LMB and IF staining was conducted using anti-p62 and anti-SIRT6 antibodies. The treatment of LMB markedly reduced the number of p62 and SIRT6 puncta and inhibited the translocation of both proteins to the cytoplasm, confirming that the two proteins overlap in the nucleus (Figure 6C). We investigated the possibility of some physical interaction between SIRT6 and p62 by co-immunoprecipitation (Co-IP). Co-IP assays for p62 and ubiquitin were performed using lysates of CK-treated cells, which were subsequently immunoblotted using anti-SIRT6 antibody (Figure 6D,E). These results suggest that relocation of nuclear SIRT6 to the cytoplasm is p62-dependent via ubiquitination.

## 4. Discussion

**Figure 7:**
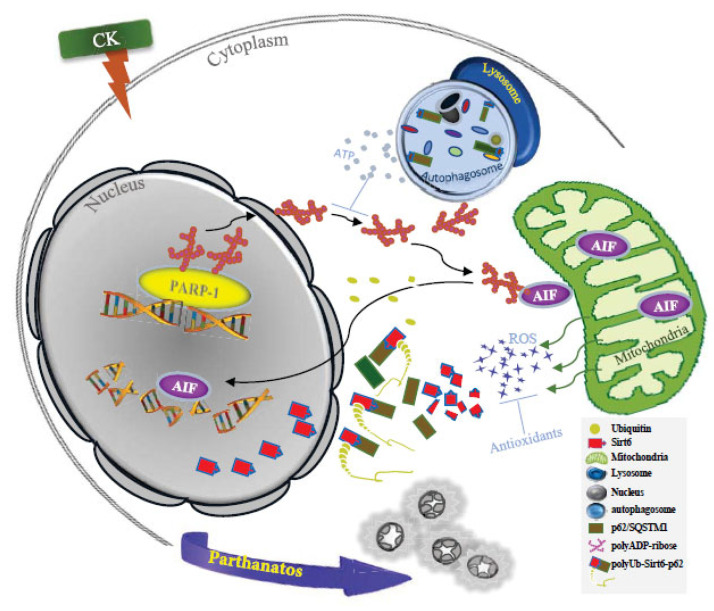
Schematic diagram of CK-induced PARP-1 activation and its regulatory mechanism.

The diverse pharmacological effects of ginsenosides on human health have been hampered by their low absorption rate, which prevents them from exerting their full effects in vivo. However, due to its relatively high absorption, CK has been investigated for a variety of pharmacological uses, with many studies focusing on its anticancer effects [3,20]. Despite the remarkable anticancer activity of CK against lung cancer cells, the molecular mechanisms underlying it require further investigation, and in this study, we identified a novel molecular mechanism induced by CK: caspase-independent PARP-1-mediated parthanatos and the role of SIRT6 in this process (Figure 7).

Despite the anti-cancer effects of CK, its effects on lung cancer remain to be investigated. CK induces caspase- and mitochondria-dependent apoptosis in H460 lung cancer cells [24] and ER stress, leading to apoptosis in A549 and SK-MES-1 human lung cancer cells [21]. CK synergizes with cisplatin-induced apoptosis in H460 and A549 cells [30]. Therefore, the anticancer effects of CK on lung cancer appear to be closely related to apoptosis. In the present study, the treatment of H460 cells with CK resulted in cell-cycle arrest via the upregulation of p27, p21, and p53. Despite decreased caspase-8 levels, CK did not significantly induce caspase-3 and PARP-1 apoptosis. However, long-term incubation (>36 h) slightly reduced caspase-3 levels and downstream PARP-1 degradation. (Figure S1). This indicates that canonical caspase-dependent apoptosis is insensitive to CK.

PARP-1 hyperactivation induces parthanatos via cytoplasmic poly (ADP-ribose) accumulation [31]. Thus, PARP-1 activation can promote cell survival or cell death. When DNA is damaged, PARP-1 is cleaved by caspases-3 and -7 into two biologically inactive fragments (89 and 24 kDa) that induce cell death [18]. Parthanatos is a caspase-independent PARP-1 hyperactivation-mediated cell-death pathway involved in various pathophysiological processes, including neurological and cardiovascular diseases [32]. The hallmarks of parthanatos include PARP-1 hyperactivation, AIF nuclear translocation, massive DNA fragmentation, chromatin condensation (distinct from apoptotic nuclear fragmentation), and the depolarization of the mitochondrial membrane [17,31,32]. When A549 cells were treated with CK, all parthanatos characteristics were observed. When CK-induced PARP-1 activation was inhibited using 3-AB or ANI, apoptotic PARP-1 cleavage was induced, indicating that CK induces different types of cell death depending on the biological context. However, the causes of PARP-1 hyperactivation remain unclear. CK-induced PAR accumulation was inhibited by NAD+ supplementation and PARP inhibitor treatment. Although PARP-1 activation depends on NAD+ levels, NAD+ supplementation inhibited CK-induced PARP-1 accumulation, suggesting that PARP-1 activation is related to cellular energy levels. NAD+ plays a critical role as a cofactor in cellular energy metabolism and redox homeostasis [33]. Therefore, its supplementation may result in cytoprotection by increasing cellular ATP levels through improved mitochondrial function [34,35]. NAD+ supplementation increased CK-induced autophagy and outer mitochondrial membrane protein Nix levels, while reducing antioxidant enzyme levels (Figure S2). Autophagy may reduce PARP-1 activation by increasing cellular energy levels via catabolism. When autophagy was reduced by autophagy blockade, SIRT6 knockdown, and proteasome inhibition, CK-induced PARylation levels further increased (Figure 3B, Figure 4D,F, Figure S3 and Figure S4). However, they decreased under autophagy-promoting conditions, such as NAD+ supplementation and SIRT6 overexpression (Figure 2F, Figure 3F and Figure S2). These results indicate that PARP-1 activation may depend on the cellular energy levels regulated by autophagy.

SIRT6 is predominantly localized in the nucleus and functions as a mono-ADP-ribosyltransferase and NAD+-dependent deacetylase [5,6]. Therefore, changes in subcellular localization and its expression levels can be critical to its function. SIRT6 protein stability is regulated by the p53 and c-Jun/c-Fos pathways and post-transcriptional modifications [36,37]. Proteasome inhibition via non-canonical ubiquitination or deubiquitination plays an important role in SIRT6 stability [38,39]. In the present study, SIRT6 was associated with ubiquitin, as demonstrated using Co-IP. However, proteasome inhibition by MG132 decreased SIRT6 protein levels instead of increasing them (Figure S3), which suggests that ubiquitinated SIRT6 is not a proteasome target. SIRT6 protein levels are upregulated by NAD+ supplementation and antioxidants, indicating that SIRT6 stability may be regulated by oxidative stress. Therefore, the decrease in SIRT6 protein levels by CK after autophagy or proteasome inhibition may be related to oxidative stress. Indeed, antioxidant enzyme levels increased under these conditions.

We found that autophagy inhibition further increased CK-induced HO-1 and SOD2 levels (Figure 4). Furthermore, the levels of the CK-induced antioxidant enzymes HO-1 and SOD2 increased after SIRT6 knockdown and decreased after SIRT6 overexpression (Figure S4). Sirtuins modulate oxidative-stress genes and related signaling pathways [40]. SIRT6 plays a protective role in response to oxidative stress, protecting against ischemia/reperfusion injury by regulating antioxidants [41] and by activating NRF2 in human mesenchymal stem cells [42]. In contrast, SIRT6 promotes ROS generation in various cancer-cell types, including lung cancer cells, leading to cell death and mitochondrial dysfunction [43,44]. In the present study, CK decreased SIRT6 protein levels and increased the levels of antioxidant enzymes, such as HO-1 and SOD2, which were recovered by antioxidant or SIRT6 overexpression, thereby increasing cell viability. These results suggest that the oxidative stress caused by decreased SIRT6 protein levels plays an important role in CK-induced cell death. Following CK treatment, SIRT6 was detected in the cytoplasmic fraction, indicating a role in the cytoplasm rather than the nucleus. Because the function of sirtuins varies depending on their subcellular location [45,46], a better understanding of SIRT6 function requires the identification of SIRT6 target organelles. We observed that LMB inhibited SIRT6 translocation from the nucleus to the cytosol after the CK treatment. However, the underlying mechanism of this translocation, which occurs in response to CK, remains unclear.

We hypothesized that this translocation depends on p62, the levels of which increase in response to CK. p62 regulates the quality of intracellular proteins by selectively targeting ubiquitinated substrates and transporting them to the autophagosome or proteasome for degradation [47,48]. In the present study, CK treatment resulted in the intracellular redistribution of SIRT6 to the cytoplasm, and IF staining confirmed its colocalization with p62. Co-IP analysis showed that SIRT6 associates with ubiquitin and p62, suggesting that it interacts with ubiquitinated p62. These results were supported by the upregulation of SIRT6 in the nucleus following p62 knockdown, indicating that p62 may be involved in SIRT6 stability and intracellular redistribution. Additionally, although total protein levels decreased after LMB treatment (Figure 6A), the size of the nuclear p62 puncta increased, which may have resulted from p62 accumulation. Fu et al. [49]. demonstrated that nuclear p62 aggregates act as active proteolytic sites for protein degradation via proteasome recruitment. Although p62 and SIRT6 accumulation was not detected upon proteasome inhibition, whether nuclear-localized p62 recruits proteasomes for SIRT6 degradation remains unclear. Nevertheless, p62 may also play an important role in the degradation of nuclear and cytoplasmic proteins. Additionally, although SIRT6 was translocated to the particulate fraction, elucidating its target organelles is key to understanding its function.

Although this study sheds light on a completely new mechanism for the pharmacological activity of CK, the health benefits of CK need to be evaluated not only through in vitro studies, but also in vivo studies. Moreover, it is necessary to evaluate whether CK is effective not only in lung cancer but also in other types of cancer cells through the same mechanism.

## 5. Conclusions

This study demonstrated a novel molecular mechanism underlying the chemopreventive effect of CK in lung cancer cells, involving PARP-1 activation-mediated parthanatos, which is promoted by SIRT6 degradation. Nuclear SIRT6 translocates to the cytoplasm in a p62-dependent manner, leading to PARP-1 activation. This suggests that PARP-1 hyperactivation is a key regulator of the chemopreventive effects of CK, which may be further promoted by SIRT6 protein regulation. Based on these conclusions, it will be very important to perform in vivo experiments to lay the foundation for a further approach.

## Data Availability

The data presented in this study are available in the article.

## Supplementary Materials

The following supporting information can be downloaded at: https://www.mdpi.com/article/10.3390/nu17030539/s1, Figure S1: Changes in expression of apoptosis and autophagy-related proteins after up to 48 h of CK exposure; Figure S2: Effects of NAD^+^ on CK-induced autophagy-related proteins and antioxidant enzymes; Figure S3: Effects of knockdown and overexpression of Sirt6 on CK-induced autophagy and antioxidant enzymes; Figure S4: Effect of MG132 on CK-induced Sirt6 and PolyADP-ribose expression; Figure S5: Densitometry data in Figure 1C,D; Figure S6: Densitometry data in Figure 2A,C,E,F; Figure S7: Densitometry data in Figure 3A,B,D,F; Figure S8: Densitometry data in Figure 4A,C,E,F,G; Figure S9: Densitometry data in Figure 5A–C and Figure 6A,B.

## Abbreviations

AIF, apoptosis-inducing factor; 3-AB, 3-aminobenzamide; ANI, 4-amino-1,8-naphthalimide; BaF1, bafilomycin A1; CK, compound K; CQ, chloroquine; H3K9Ac, acetylated histone H3 lysine 9; HO-1, heme-oxygenase 1; IF, immunofluorescence; IP, immunoprecipitation; NAC, *N*-acetyl cysteine; NAD+, nicotinamide adenine dinucleotide; PARP-1, poly (ADP-ribose) polymerase 1; PARylation, ADP ribosylation; ROS, reactive oxygen species; SIRT6, silent information regulator 6; SOD2, superoxide dismutase 2.
