# Supplementary material for: The Chemopreventive Effect of Ginsenoside Compound K Is Regulated by PARP-1 Hyperactivation, Which Is Promoted by p62-Dependent SIRT6 Degradation
Source: Nutrients. 2025 Jan 31;17(3):539. doi: 10.3390/nu17030539 (PMC11821008; doi:10.3390/nu17030539)
Supplement: Supplementary file 1 [file nutrients-17-00539-s001.zip › nutrients-3454048-supplementary.pptx]

## Slide 1
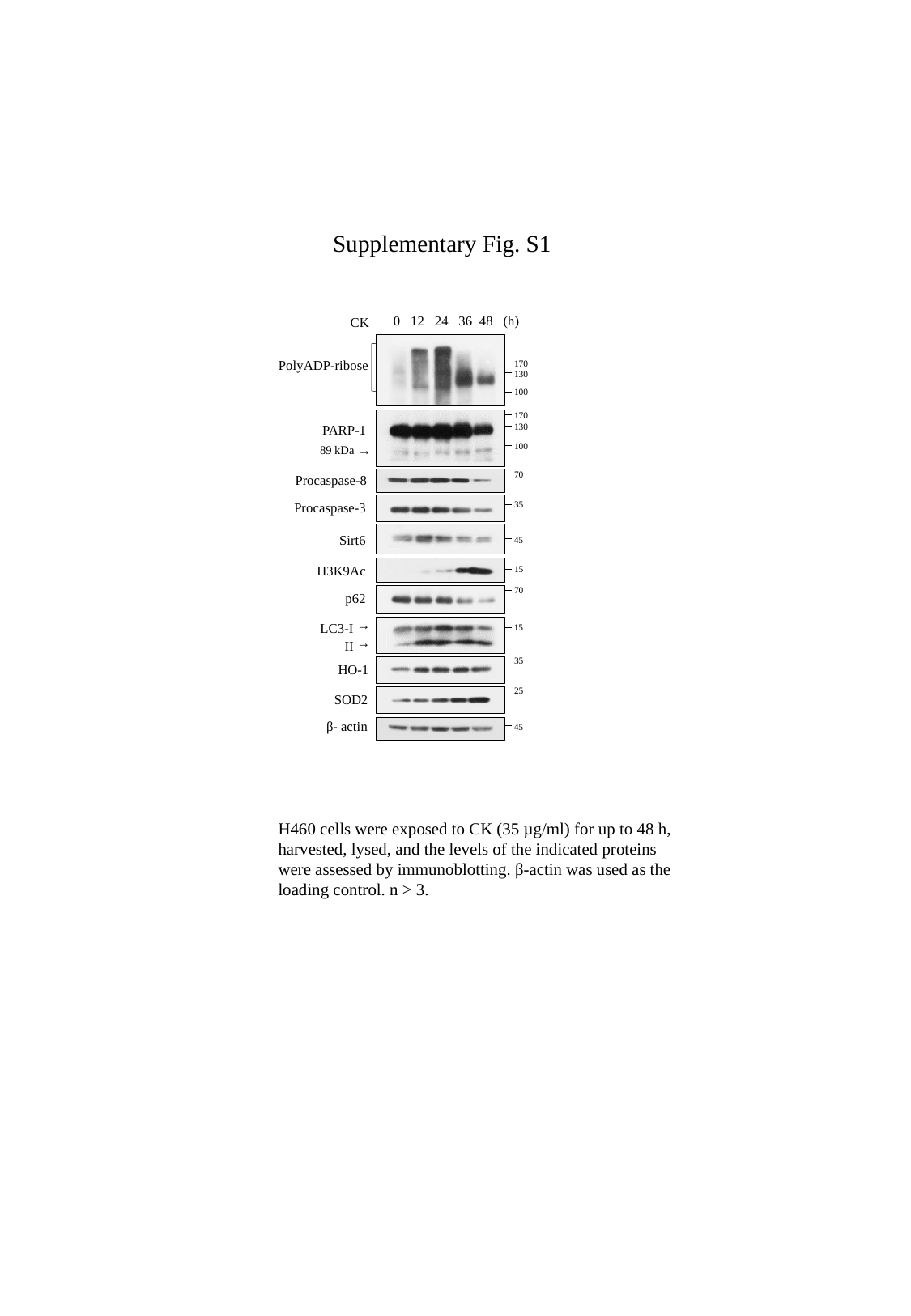

Supplementary Fig. S1
0 12 24 36 48 (h)
CK
PolyADP-ribose
170
130
100
170
130
PARP-1
100
89 kDa
→
70
Procaspase-8
35
Procaspase-3
Sirt6
45
H3K9Ac
15
70
p62
→
LC3-I
15
→
II
35
HO-1
25
SOD2
β- actin
45
H460 cells were exposed to CK (35 µg/ml) for up to 48 h, harvested, lysed, and the levels of the indicated proteins were assessed by immunoblotting. β-actin was used as the loading control. n > 3.

## Slide 2
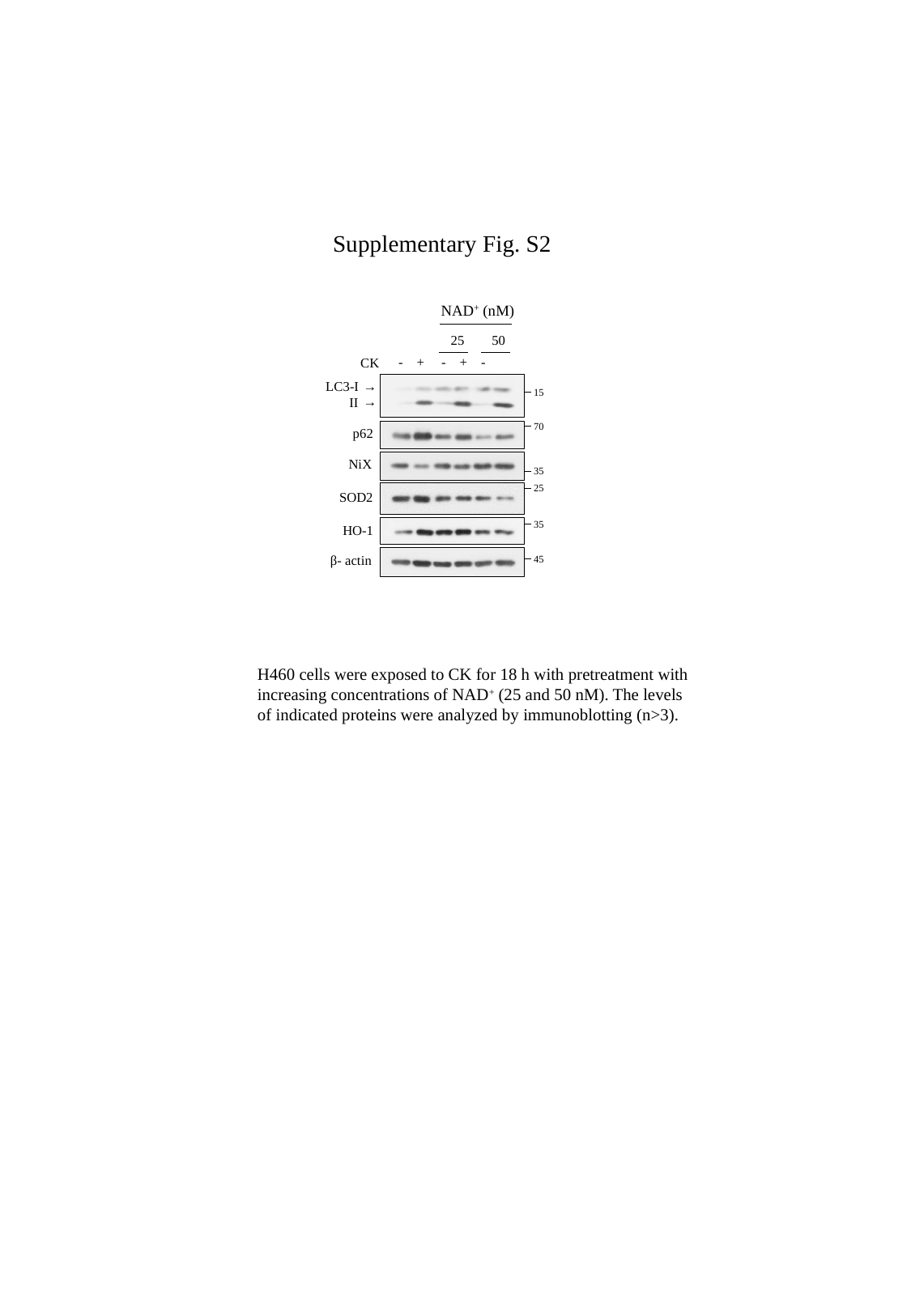

Supplementary Fig. S2
NAD+ (nM)
25
50
- + - + - +
CK
LC3-I
→
15
II
→
70
p62
NiX
35
25
SOD2
35
HO-1
β- actin
45
H460 cells were exposed to CK for 18 h with pretreatment with increasing concentrations of NAD+ (25 and 50 nM). The levels of indicated proteins were analyzed by immunoblotting (n>3).

## Slide 3
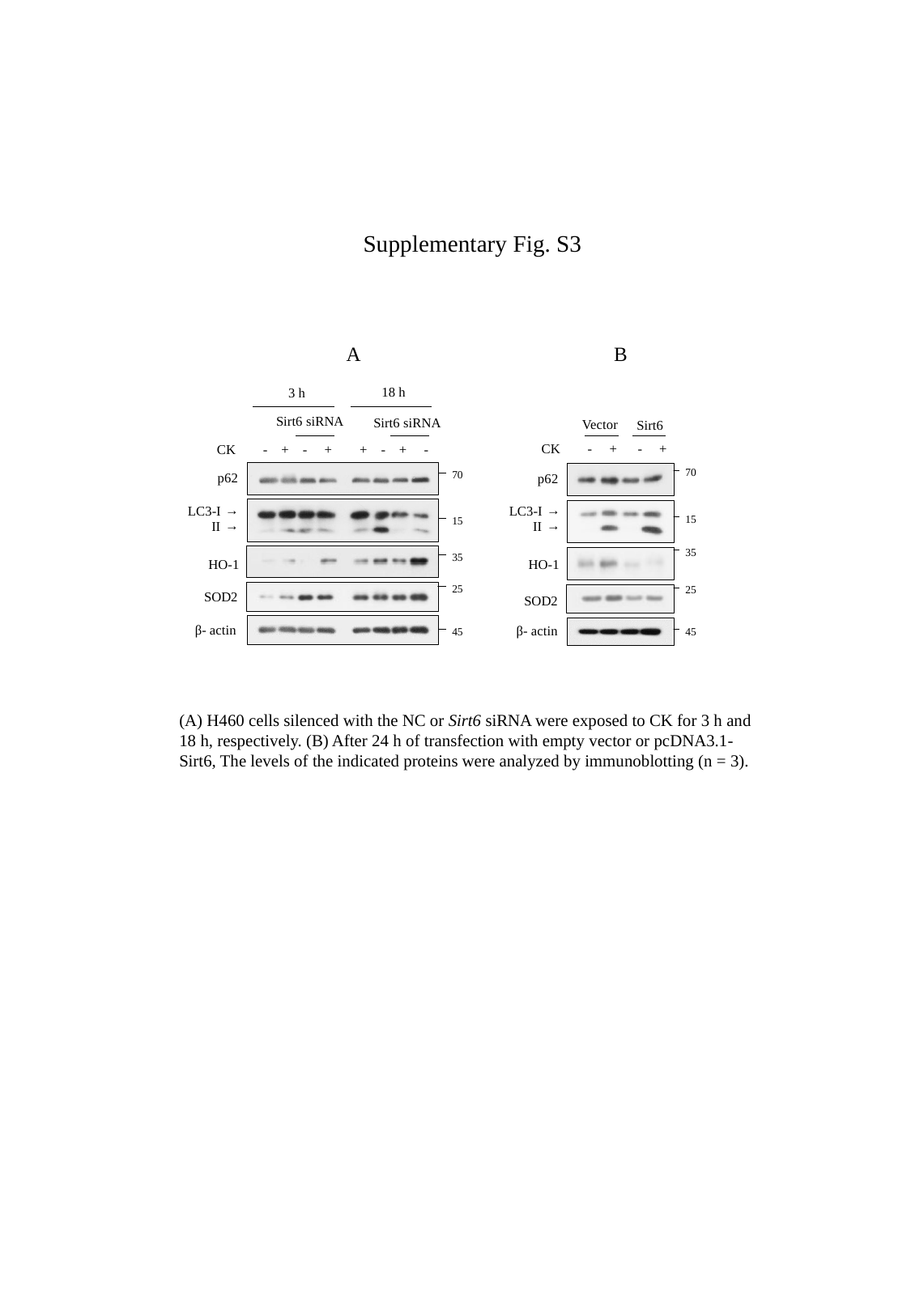

Supplementary Fig. S3
A
B
18 h
3 h
 Sirt6 siRNA
 Sirt6 siRNA
Vector
Sirt6
CK - + - + + - + -
CK - + - +
70
70
p62
p62
LC3-I
LC3-I
→
→
15
15
II
II
→
→
35
35
HO-1
HO-1
25
25
SOD2
SOD2
β- actin
β- actin
45
45
(A) H460 cells silenced with the NC or Sirt6 siRNA were exposed to CK for 3 h and 18 h, respectively. (B) After 24 h of transfection with empty vector or pcDNA3.1-Sirt6, The levels of the indicated proteins were analyzed by immunoblotting (n = 3).

## Slide 4
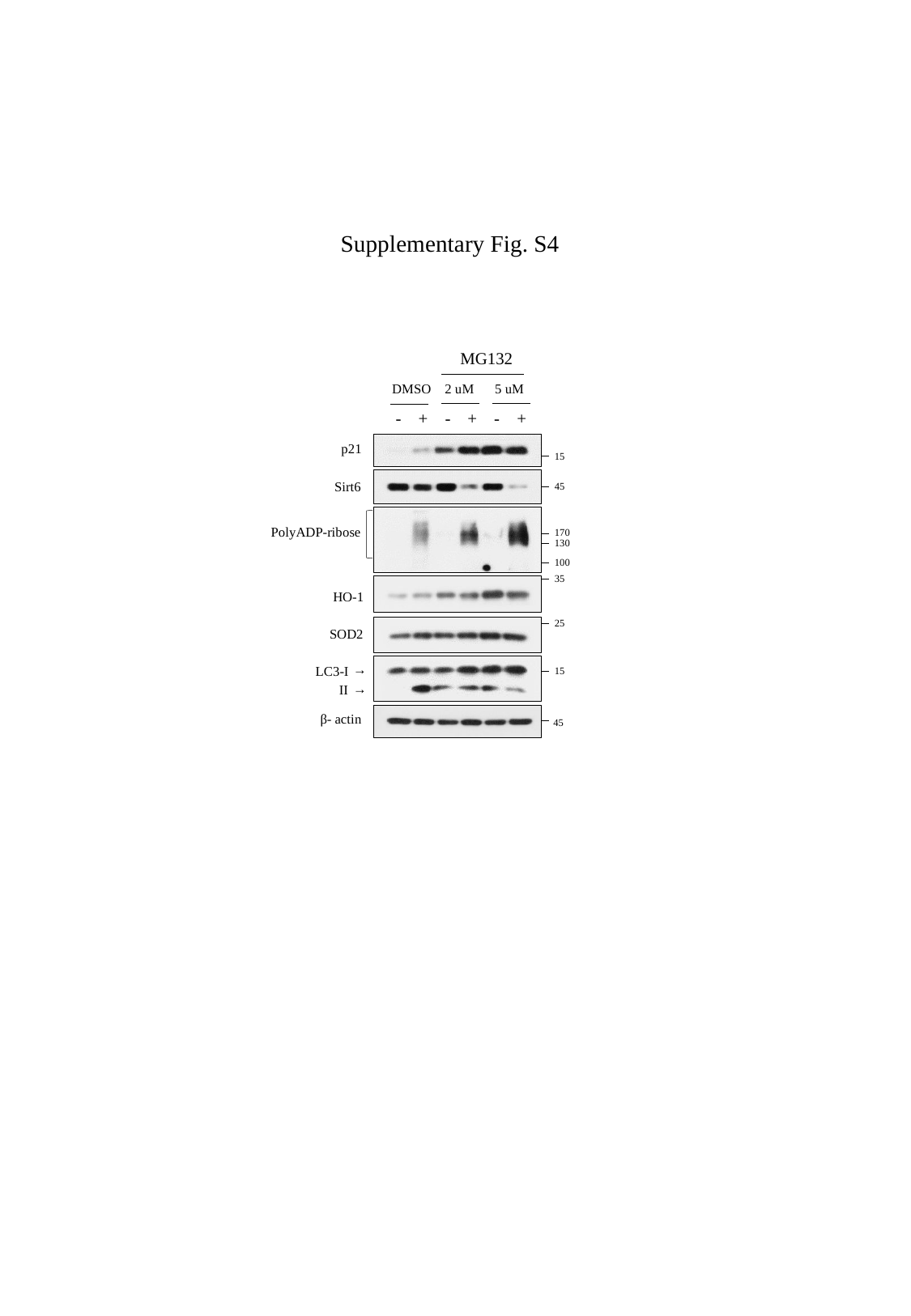

Supplementary Fig. S4
MG132
DMSO 2 uM 5 uM
- + - + - +
p21
15
Sirt6
45
PolyADP-ribose
170
130
100
35
HO-1
25
SOD2
→
LC3-I
15
II
→
β- actin
45

## Slide 5
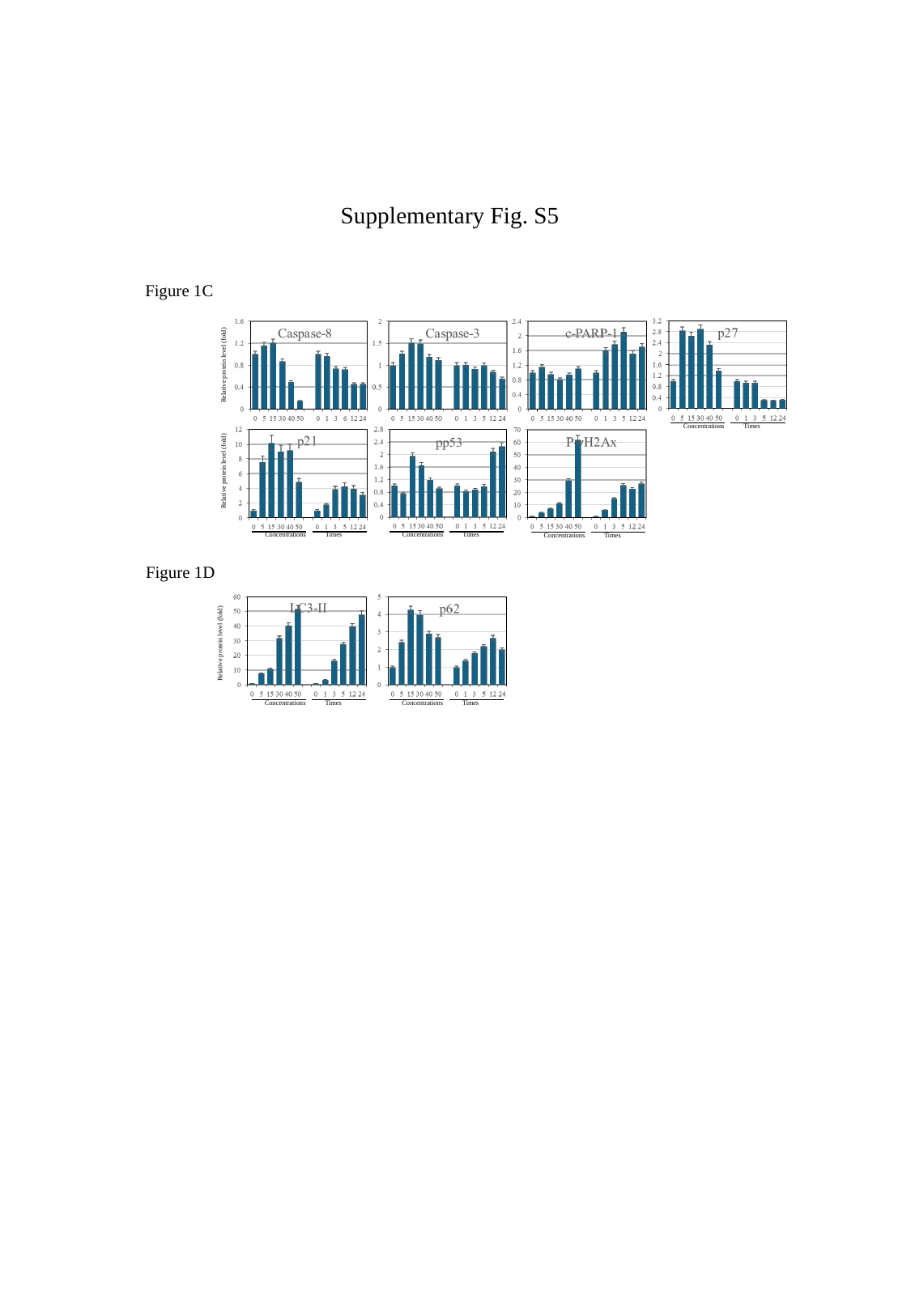

Supplementary Fig. S5
Figure 1C
Relative protein level (fold)
Concentrations Times
Relative protein level (fold)
Concentrations Times
Concentrations Times
Concentrations Times
Figure 1D
Relative protein level (fold)
Concentrations Times
Concentrations Times

## Slide 6
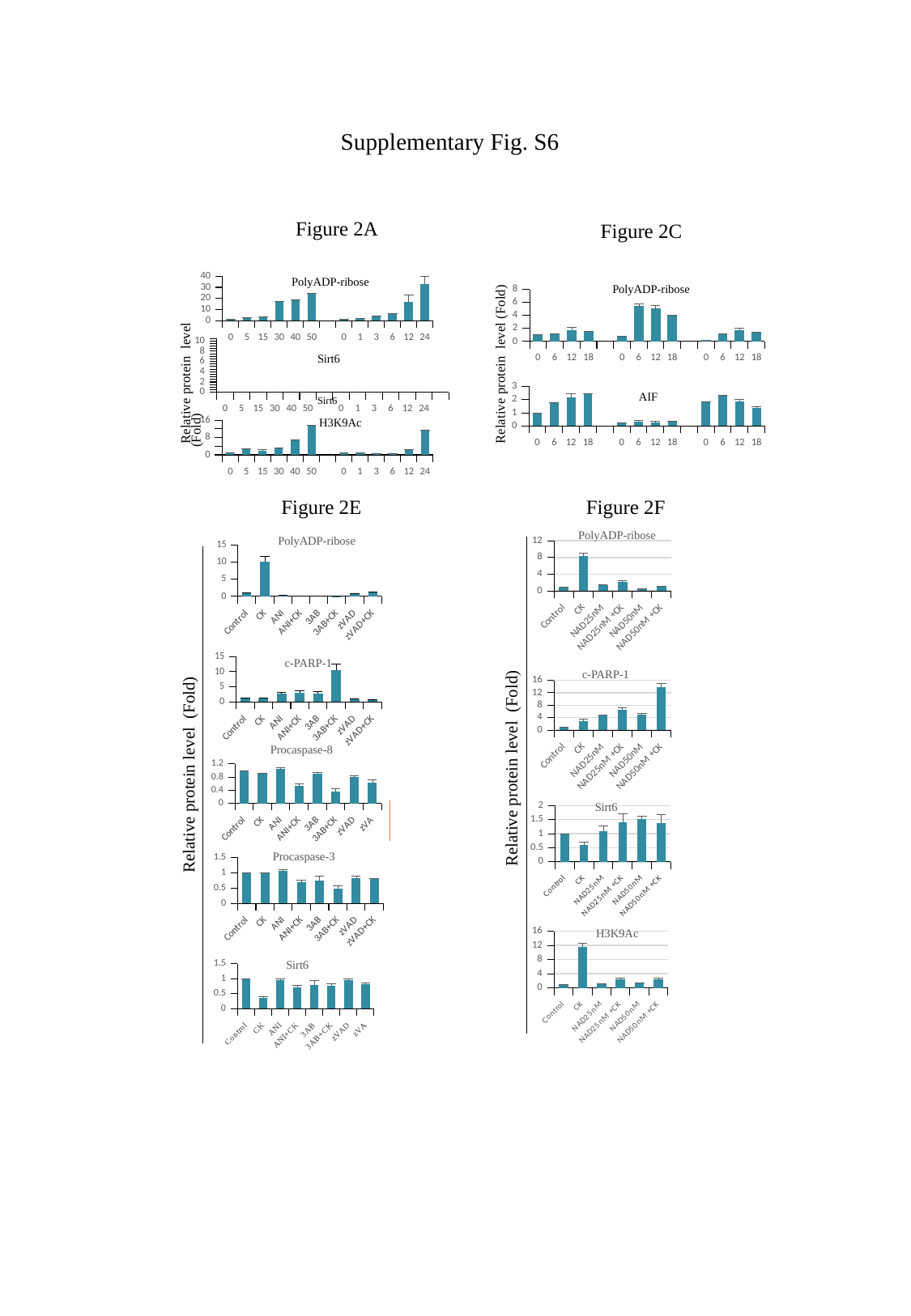

Supplementary Fig. S6
Figure 2A
Figure 2C
### Chart
| Category | |
|---|---|
| 0 | 1.0 |
| 5 | 2.356027509159221 |
| 15 | 3.070002763514296 |
| 30 | 16.629257793211917 |
| 40 | 18.510760433790466 |
| 50 | 24.76384754341867 |
| | None |
| 0 | 1.0 |
| 1 | 1.926533820861829 |
| 3 | 3.98234200212802 |
| 6 | 5.71312177489812 |
| 12 | 16.89725546328214 |
| 24 | 32.67228777378271 |PolyADP-ribose
### Chart
| Category | |
|---|---|
| 0 | 1.0 |
| 6 | 1.1835428074356504 |
| 12 | 1.668980719676805 |
| 18 | 1.5145645450360103 |
| | None |
| 0 | 0.7947824282918254 |
| 6 | 5.351662514745857 |
| 12 | 4.9518133213558615 |
| 18 | 4.007683905694616 |
| | None |
| 0 | 0.11248537261887648 |
| 6 | 1.1071834460960273 |
| 12 | 1.6984375052107439 |
| 18 | 1.430774728589349 |PolyADP-ribose
### Chart
| Category | |
|---|---|
| 0 | 1.0 |
| 5 | 1.3108072833095112 |
| 15 | 1.7967023695412785 |
| 30 | 1.8583374161077972 |
| 40 | 1.2907714891809605 |
| 50 | 0.8822327024878357 |
| | None |
| 0 | 1.0 |
| 1 | 1.221858020080042 |
| 3 | 1.284755400981286 |
| 6 | 1.3923334970448136 |
| 12 | 0.9202025328860511 |
| 24 | 0.719738307523928 |Sirt6
 Relative protein level (Fold)
 Relative protein level (Fold)
### Chart
| Category | |
|---|---|
| 0 | 1.0 |
| 6 | 1.741931609861791 |
| 12 | 2.1705416252229797 |
| 18 | 2.4169886964916145 |
| | None |
| 0 | 0.2719616669086685 |
| 6 | 0.3064425353033259 |
| 12 | 0.28078859681754326 |
| 18 | 0.3292483143909357 |
| | None |
| 0 | 1.809372710310408 |
| 6 | 2.2722313559900056 |
| 12 | 1.8292901590893589 |
| 18 | 1.3923704142918056 |AIF
AIF
Sirt6
### Chart
| Category | |
|---|---|
| 0 | 1.0 |
| 5 | 2.5924818317903946 |
| 15 | 2.0164279083770293 |
| 30 | 2.846711324084063 |
| 40 | 6.740426670680817 |
| 50 | 13.56140896660729 |
| | None |
| 0 | 1.0 |
| 1 | 1.0784374084280255 |
| 3 | 0.6536618430127069 |
| 6 | 0.7433557595257053 |
| 12 | 2.2494383585566333 |
| 24 | 11.386687543843552 |H3K9Ac
Figure 2E
Figure 2F
### Chart
| Category | |
|---|---|
| Control | 1.0 |
| CK | 8.436919638047371 |
| NAD25nM | 1.3531042919818528 |
| NAD25nM +CK | 2.099706705282679 |
| NAD50nM | 0.4383802372405049 |
| NAD50nM +CK | 1.094438499224955 |PolyADP-ribose
### Chart
| Category | |
|---|---|
| Control | 1.0 |
| CK | 10.1961139605794 |
| ANI | 0.21323078498618073 |
| ANI+CK | 0.19297063875908127 |
| 3AB | 0.05027699513917421 |
| 3AB+CK | 0.02717496656429713 |
| zVAD | 0.7503925723363297 |
| zVAD+CK | 1.1770007473504416 |PolyADP-ribose
### Chart
| Category | |
|---|---|
| Control | 1.0 |
| CK | 1.1134554596055615 |
| ANI | 2.615788991456124 |
| ANI+CK | 3.0662350825755325 |
| 3AB | 2.829417390109261 |
| 3AB+CK | 10.462650605859313 |
| zVAD | 0.929634709210436 |
| zVAD+CK | 0.5628264081361524 |c-PARP-1
### Chart
| Category | |
|---|---|
| Control | 1.0 |
| CK | 2.9955784923553592 |
| NAD25nM | 4.712543143837906 |
| NAD25nM +CK | 6.454468830490038 |
| NAD50nM | 4.876042397502483 |
| NAD50nM +CK | 13.800038611674852 |c-PARP-1
 Relative protein level (Fold)
Procaspase-8
 Relative protein level (Fold)
### Chart
| Category | |
|---|---|
| Control | 1.0 |
| CK | 0.9093870532506196 |
| ANI | 1.044970256863653 |
| ANI+CK | 0.529970015929583 |
| 3AB | 0.9080944768832491 |
| 3AB+CK | 0.3726432981569591 |
| zVAD | 0.8061868726548211 |
| zVAD+CK | 0.6359667776360108 |
Sirt6
### Chart
| Category | |
|---|---|
| Control | 1.0 |
| CK | 0.6047054347412963 |
| NAD25nM | 1.08669942957229 |
| NAD25nM +CK | 1.4175525491879084 |
| NAD50nM | 1.5329264826052804 |
| NAD50nM +CK | 1.374212435229058 |Procaspase-3
### Chart
| Category | |
|---|---|
| Control | 1.0 |
| CK | 0.9772738501400541 |
| ANI | 1.0626661893672655 |
| ANI+CK | 0.6921954993040579 |
| 3AB | 0.7414870002603362 |
| 3AB+CK | 0.48777970369412316 |
| zVAD | 0.8251350573703378 |
| zVAD+CK | 0.7911293338865518 |H3K9Ac
### Chart
| Category | |
|---|---|
| Control | 1.0 |
| CK | 11.522052633365744 |
| NAD25nM | 1.0168246572949253 |
| NAD25nM +CK | 2.449641035578312 |
| NAD50nM | 1.3936650151111796 |
| NAD50nM +CK | 2.4989878198895394 |Sirt6
### Chart
| Category | |
|---|---|
| Control | 1.0 |
| CK | 0.3561917379318478 |
| ANI | 0.9547321403860893 |
| ANI+CK | 0.7164228188161738 |
| 3AB | 0.7897796886111682 |
| 3AB+CK | 0.7612978024212714 |
| zVAD | 0.9562605664393641 |
| zVAD+CK | 0.811218685736495 |

## Slide 7
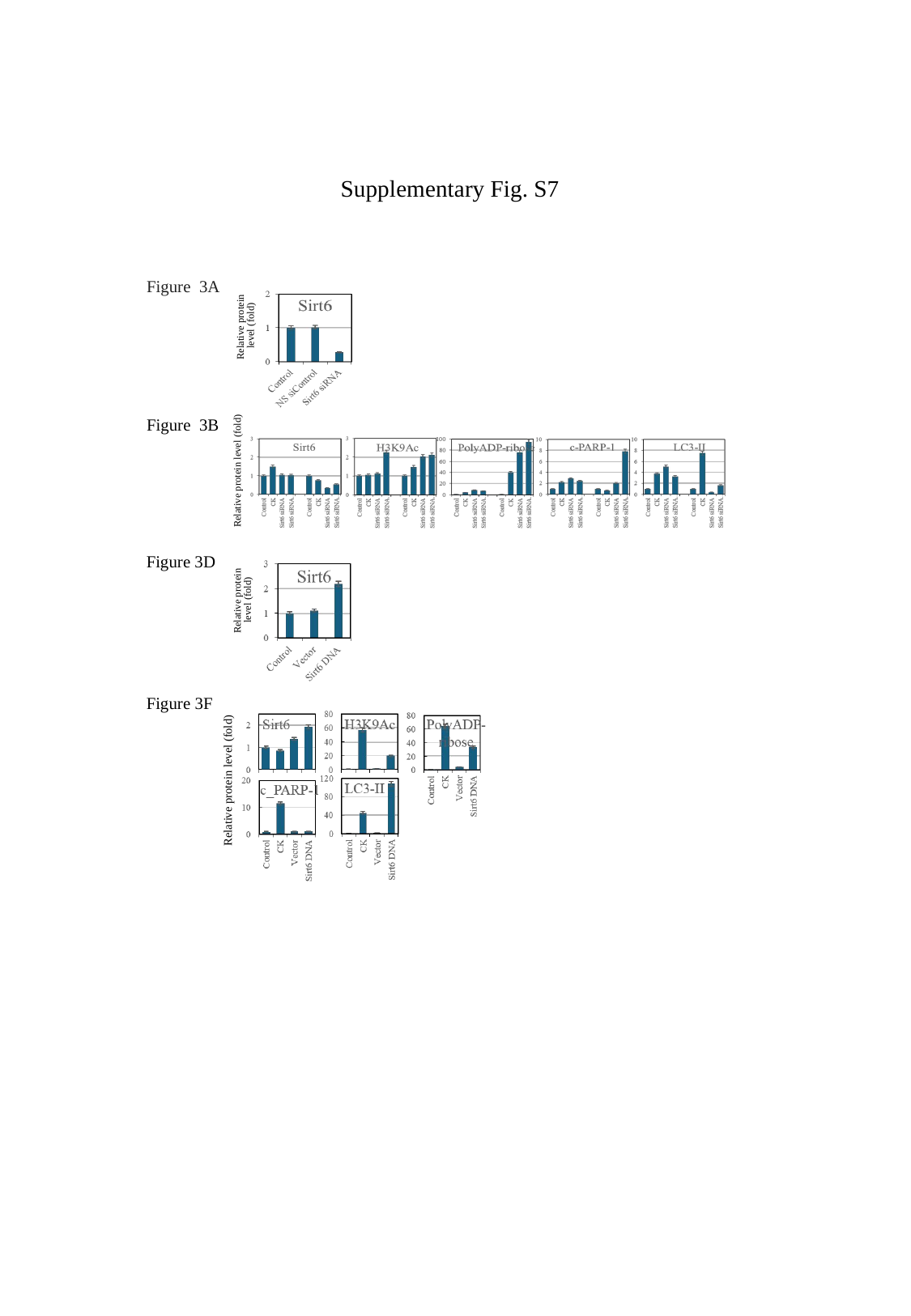

Supplementary Fig. S7
Figure 3A
Relative protein
level (fold)
Figure 3B
Relative protein level (fold)
Figure 3D
Relative protein
level (fold)
Figure 3F
Relative protein level (fold)

## Slide 8
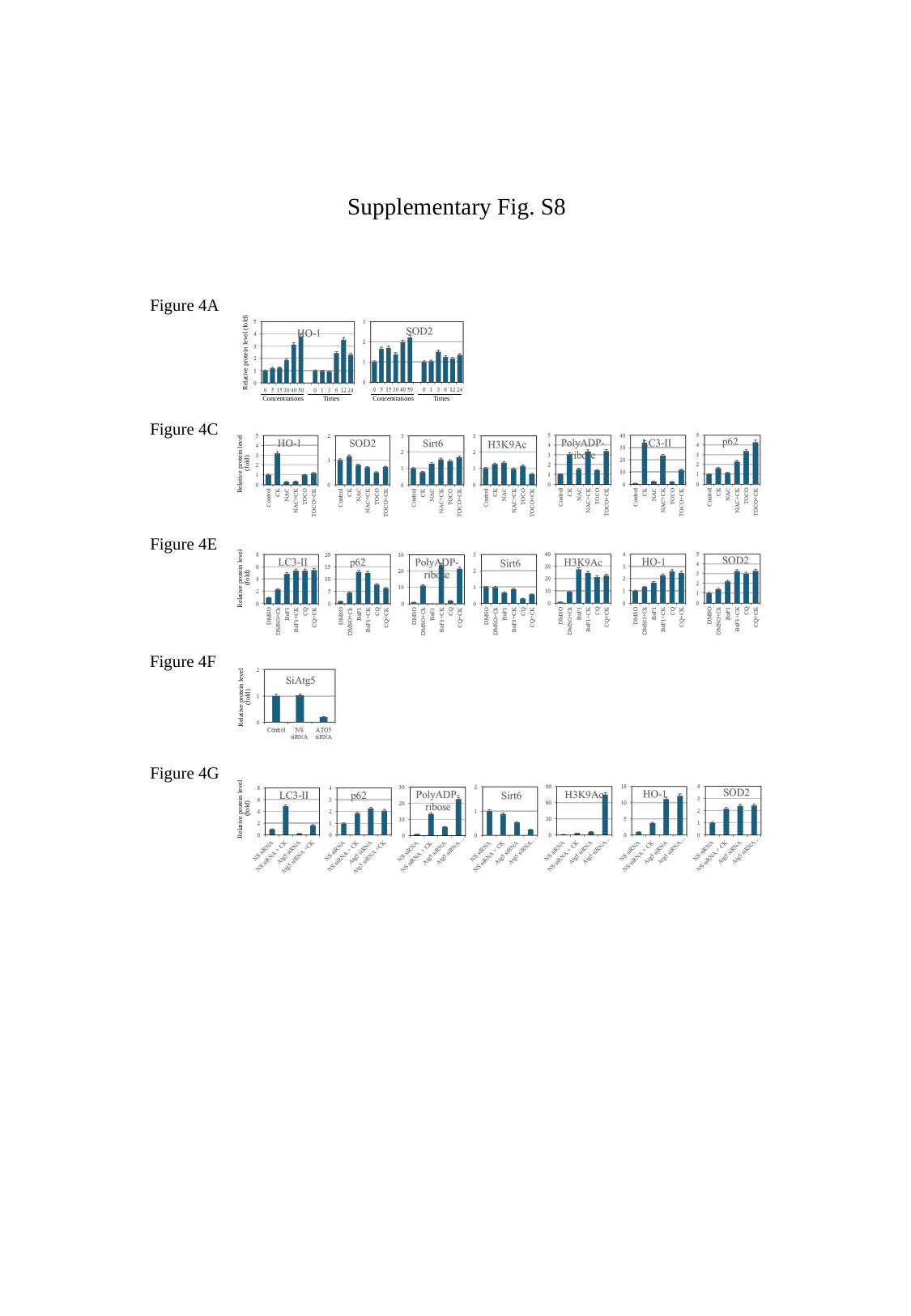

Supplementary Fig. S8
Figure 4A
Relative protein level (fold)
Concentrations Times
Concentrations Times
Figure 4C
Relative protein level
(fold)
Figure 4E
Relative protein level
(fold)
Figure 4F
Relative protein level
(fold)
Figure 4G
Relative protein level
(fold)

## Slide 9
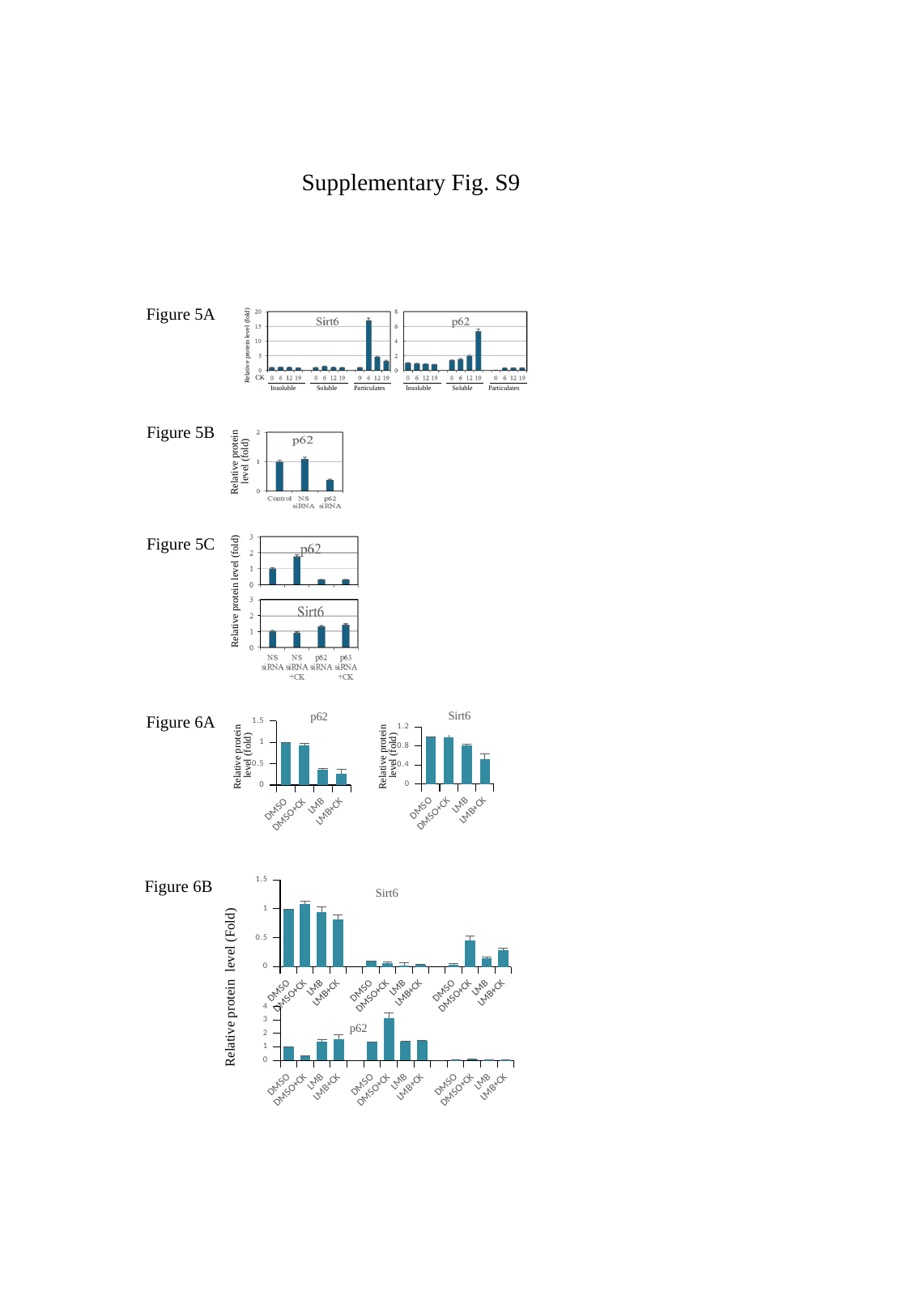

Supplementary Fig. S9
Relative protein level (fold)
CK
Insoluble
Soluble
Insoluble
Soluble
Particulates
Particulates
Figure 5A
Figure 5B
Relative protein
level (fold)
Relative protein level (fold)
Figure 5C
### Chart
| Category | |
|---|---|
| DMSO | 1.0 |
| DMSO+CK | 0.9277666881928802 |
| LMB | 0.35391535310401145 |
| LMB+CK | 0.26527542395340376 |
Sirt6
p62
Figure 6A
### Chart
| Category | |
|---|---|
| DMSO | 1.0 |
| DMSO+CK | 0.9924755617858958 |
| LMB | 0.8208498540029552 |
| LMB+CK | 0.5234226587893934 |
Relative protein
level (fold)
Relative protein
level (fold)
### Chart
| Category | |
|---|---|
| DMSO | 1.0 |
| DMSO+CK | 1.087230266609189 |
| LMB | 0.9433547218770157 |
| LMB+CK | 0.8240657814611316 |
| | None |
| DMSO | 0.09808550635378077 |
| DMSO+CK | 0.06263949992792171 |
| LMB | 0.019408969147302075 |
| LMB+CK | 0.031235982368776935 |
| | None |
| DMSO | 0.038149462479124006 |
| DMSO+CK | 0.4565170911562093 |
| LMB | 0.1493365675834183 |
| LMB+CK | 0.28286370987983117 |Figure 6B
Sirt6
 Relative protein level (Fold)
### Chart
| Category | |
|---|---|
| DMSO | 1.0 |
| DMSO+CK | 0.3241955250948569 |
| LMB | 1.3784008654969775 |
| LMB+CK | 1.567065680680822 |
| | 0.0 |
| DMSO | 1.397696744131536 |
| DMSO+CK | 3.1616407115907115 |
| LMB | 1.3891914823762956 |
| LMB+CK | 1.4715475494543182 |
| | 0.0 |
| DMSO | 0.020325572798735227 |
| DMSO+CK | 0.045466084611520734 |
| LMB | 0.0076680206613120964 |
| LMB+CK | 0.008112529056098964 |p62
